# Conformation and Dynamics of Long-Chain End-Tethered Polymers in Microchannels

**DOI:** 10.3390/polym11030488

**Published:** 2019-03-13

**Authors:** Tamal Roy, Kai Szuttor, Jens Smiatek, Christian Holm, Steffen Hardt

**Affiliations:** 1 Institute for Nano- and Microfluidics, Technische Universität Darmstadt, 64287 Darmstadt, Germany; tamalroy@uic.edu; 2 Institut für Computerphysik, Universität Stuttgart, 70569 Stuttgart, Germany; kai@icp.uni-stuttgart.de (K.S.); jenssmiatek@googlemail.com (J.S.); holm@icp.uni-stuttgart.de (C.H.)

**Keywords:** polyelectrolyte, confinement, DNA, stretching, relaxation, Lattice Boltzmann, molecular dynamics

## Abstract

Polyelectrolytes constitute an important group of materials, used for such different purposes as the stabilization of emulsions and suspensions or oil recovery. They are also studied and utilized in the field of microfluidics. With respect to the latter, a part of the interest in polyelectrolytes inside microchannels stems from genetic analysis, considering that deoxyribonucleic acid (DNA) molecules are polyelectrolytes. This review summarizes the single-molecule experimental and molecular dynamics simulation-based studies of end-tethered polyelectrolytes, especially addressing their relaxation dynamics and deformation characteristics under various external forces in micro-confined environments. In most of these studies, DNA is considered as a model polyelectrolyte. Apart from summarizing the results obtained in that area, the most important experimental and simulation techniques are explained.

## 1. Introduction

Understanding the behavior of a linear polymer in solution is essential for designing a number of next-generation analytical devices, e.g., enabling genome sequence determination by nanopore sequencing or fluorescence in situ hybridization (FISH) assays. In the last three decades, the development of micro and nanofabrication techniques has facilitated the fabrication of lab-on-a-chip devices. Furthermore, the development of advanced fluorescence visualization methods, e.g., laser-induced epifluorescence and confocal microscopy, helped scientists to observe and analyze the dynamics of biopolymers down to the single-molecule level. Experiments with micro and nanofluidic devices promoted the understanding of the dynamics of long-chain polymers exposed to different types of external forces, thereby driving the molecules away from their equilibrium coiled state. Such manipulations of long-chain polymers are of paramount importance in many practical situations such as size-based separation and sequencing of DNA molecules. Furthermore, DNA plays a key role in many approaches to configure, form, and assemble structures on the nanoscale. Examples are crystal-like networks of DNA strands [1], twisted and curved nanobundles [2], the formation of colloidal crystals mediated by DNA strands [3], or molecular motors [4,5]. A better understanding of the fundamental aspects of polymer conformation and dynamics in micro-geometries is therefore desirable.

In microchannels, long-chain polymers are often deformed by applying an external force. In most of the cases, the external force is either the hydrodynamic drag force, the electric force (in case of a polyelectrolyte which is a polymer possessing ionizable groups in solution), or a combination of both. While it is possible to deform a free polymer molecule, an easier way is to tether one end of the polymer to a surface and apply the external force. Apart from the fundamental interest of polymer physicists to understand the polymer-surface interaction, surface-tethered polymers are relevant in many well-known applications, particularly in the fields of genomics and surface functionalization. In that context it is relevant that the physical behavior of a polymer significantly changes in a confined environment. This review summarizes the experimental, numerical simulation and theoretical works aiming at understanding the conformation and dynamics of surface-tethered long-chain polymers in micro-confinement.

## 2. Theoretical Models

### 2.1. Deformation Under External Forces

**Figure 1:**
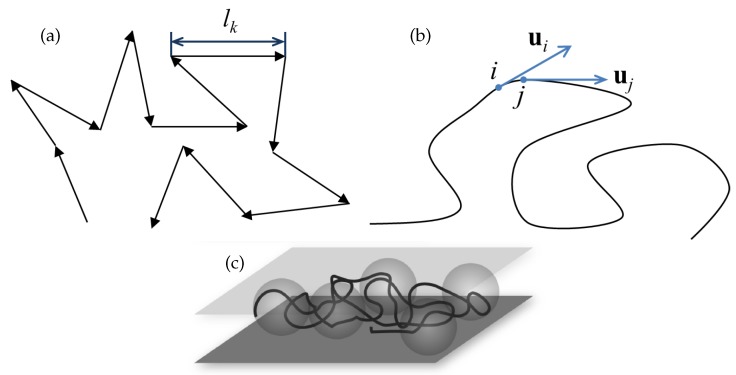
Different representations of a polymer. (**a**) Freely jointed chain model, where the black arrows represent the chain segments. (**b**) Worm-like chain model, where $u_{i}$ and $u_{i}$ are the tangents on the chain at points *i* and *j*. The tangent-tangent correlation along the chain is given by the relation $\langle u_{i}.u_{j}\rangle =\exp\left(-\frac{l_{ij}}{l_{p}}\right)$, where $l_{ij}$ is the distance between the points *i* and *j* along the chain contour. (**c**) Chain under 2D confinement.

The deformation of a linear long-chain polymer has been addressed theoretically based on different models, notably the freely-jointed chain model (see Figure 1a) and the worm-like chain model (see Figure 1b) [6]. The behavior of different polymers can be predicted with reasonable accuracy using these models. For example, the deformation of long-chain double-stranded DNA molecules can be predicted by the worm-like chain model. The deformation is characterized by the fractional extension, which is the ratio of the extended length of the molecule under the applied force and the molecular contour length. For a freely-jointed chain (alternatively known as an ideal chain) in 3D, the theoretical force-extension relationship is given by [6]

(1) $$\frac{x}{L}=\coth\left(\frac{Fl_{k}}{k_{B}T}\right)-{\left(\frac{Fl_{k}}{k_{B}T}\right)}^{-1},$$

where *x* is the average end-to-end distance of the extended molecule, *L* is the molecular contour length, *F* the applied force, $l_{k}$ the Kuhn length (i.e., the segment length), $k_{B}$ the Boltzmann constant and *T* the absolute temperature. A chain under strong confinement can be thought of as a chain confined in 2D (i.e., with one degree of freedom being eliminated, see Figure 1c). For such a condition, the force-extension behavior of a freely jointed chain is given by [7]

(2) $$\frac{x}{L}=\frac{I_{1}\left(\frac{Fl_{k}}{k_{B}T}\right)}{I_{0}\left(\frac{Fl_{k}}{k_{B}T}\right)},$$

where $I_{0}\left(x\right)$ and $I_{1}\left(x\right)$ are the modified Bessel functions of the first kind [8]. For a worm-like chain in 3D, Marko and Siggia [9] derived the force-extension relationship in the limits of small and large extension and subsequently proposed an interpolation relation given by

(3) $$\frac{Fl_{p}}{k_{B}T}=\frac{x}{L}+\frac{1}{4{\left(1-x/L\right)}^{2}}-\frac{1}{4},$$

where $l_{p}$ is the persistence length of the molecule. When a worm-like chain is confined to a quasi 2D scenario, the force-extension relationship changes to [7]

(4) $$\frac{Fl_{p}}{k_{B}T}=\frac{7x}{8L}+\frac{1}{16{\left(1-x/L\right)}^{2}}-\frac{1}{16}.$$

### 2.2. Relaxation Dynamics

An important property which dictates the dynamics of a polymer chain is its relaxation time. Stress relaxation of a polymeric chain influences the response of the chain to external forces. A brief description of the theoretical models describing the relaxation dynamics is presented in this section. The relaxation time is the characteristic time a chain takes to relax from a strained configuration to its equilibrium state. When the molecule is relaxed from a weakly stressed condition (a reasonably good approximation for most applications), the relaxation occurs with the slowest mode, corresponding to the longest relaxation time. The longest relaxation time τ is the characteristic time taken by a randomly coiled chain to diffuse within a distance equal to its radius of gyration [6]. i.e.,

(5) $$\tau ∼\frac{R_{g}^{2}}{D},$$

where $R_{g}$ is the radius of gyration and *D* the diffusion coefficient of the molecule. Rouse [10] proposed a model for the polymer dynamics where the chain is represented by *N* beads connected by (N−1) springs. For a Rouse chain, the diffusion constant is given by the equation

(6) $$D_{R}=\frac{k_{B}T}{\zeta_{R}}=\frac{k_{B}T}{6\pi \eta Nl_{k}},$$

where η is the viscosity of the solvent. The relationship between the coil radius and the chain length is expressed as $R_{g}∼bN^{\nu }$, according to the ideal chain model [6]. Using Equations (5) and (6), one can derive the scaling of the Rouse relaxation time with the chain length as

(7) $$\tau_{R}∼N^{2\nu +1}.$$

However, there are situations where hydrodynamic interactions among different segments of the chain are important. A corresponding model taking hydrodynamic interactions into account was proposed by Zimm [11]. According to this model, the diffusivity of the coil is given by the equation

(8) $$D_{Z}=\frac{k_{B}T}{\zeta_{Z}}=\frac{k_{B}T}{6\pi \eta R_{g}}∼\frac{k_{B}T}{\eta bN^{\nu }}.$$

The Zimm relaxation time, therefore, can be expressed as

(9) $$\tau_{Z}∼N^{3\nu }.$$

## 3. Double-Stranded DNA as a Model Polymer

Among the several forms of polyelectrolytes, probably the most well studied ones are nucleic acids, which are responsible for storing the genetic information or the ‘code of life’. Deoxyribonucleic acid (DNA), occurring in the chromosomes of the nucleus of a cell, carries the genetic information of all organisms. After the revolutionary discovery of the structure of DNA by Watson et al. [12] in 1953, DNA molecules have become a focal point in the fields of molecular biology, drug discovery and polymer science. During the last 60 years, numerous studies have been conducted to understand the behavior of DNA molecules in various contexts. This includes the fundamental understanding of the mechanical and chemical properties of the molecules as well as exploring different methods to manipulate them for biomedical applications. Apart from that, double-stranded DNA molecules can mimic the behavior of a variety of polymers and polyelectrolytes. In experiments, double-stranded DNA molecules are extensively used as a model polymer due to their physiological relevance, well-documented properties, versatility as a building block for macromolecular structures, and excellent chemical modifiability.

**Figure 2:**
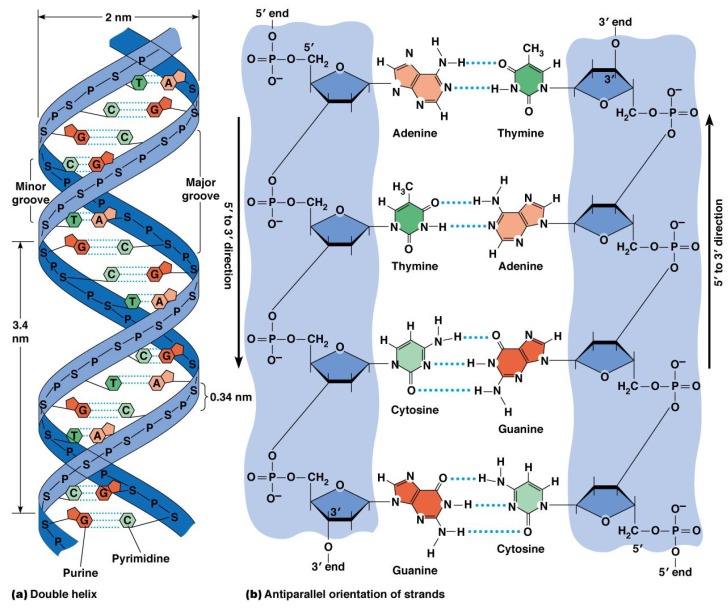
Structure of a DNA molecule [13].

DNA molecules are composed of four different types of nucleobases. These are adenine (A), thymine (T), guanine (G) and cytosine (C). An ‘A’ always makes a complementary pair with a ‘T’, and a ‘G’ always makes a complementary pair with a ‘C’. In living organisms, DNA exists in double helix form, i.e., two strands (composed of nuleobases connected by a sugar-phosphate backbone) are wound to form a twisted structure, as depicted in Figure 2. The strands are connected by hydrogen bonding between the complementary bases. The phosphate groups on the backbones of the molecule give rise to a negative charge of $2e^{-}$ per base pair in aqeuous media. Due to the high charge density on the backbone, DNA molecules are stiff over a comparatively large length scale of ∼50 nm, the persistence length of the molecules. However, at a larger length scale, the molecules behave like flexible strings.

## 4. The Role of Microchannels in Science and Technology

The advancement of microfabrication techniques and the corresponding development of microfluidics starting around 30 years ago opened up new avenues in science and technology. Microfluidic channels or microchannels are characterized by a cross-sectional dimension between one micrometer and some hundred micrometers. Microchannels are both used as a research tool for fundamental studies and as building blocks of microfluidic systems enabling novel types of applications. Naturally, the main focus of this work is on their use for soft matter research. A major portion of the different types of soft matter consists of polymers and polyelectrolytes. Researchers have extensively used microfluidic channels for studying and manipulating polyelectrolytes. The characteristic dimension of a polyelectrolyte can be comparable to the scale of the microchannel cross-section. Furthermore, the characteristic dimensions of microfluidic channels often resemble the dimension of different biological flow paths, for example arteries and veins. Therefore, it is very convenient to use such artificial channels to understand different in-vivo mechanisms. Most relevant for the thematic scope of this review is the behavior of a single polyelectrolyte in a microchannel.

Microfluidic channels often appear as building blocks of lab-on-a-chip systems, which have revolutionized bioanalytics and chemical analytics in the past decades. Lab-on-a-chip systems allow implementing analytical protocols with short analysis times, low reagent consumption and a high degree of parallelization. Naturally, in many analytical protocols conducted in lab-on-a-chip systems, especially in those including genetic analysis, DNA molecules play a key role. Therefore, in part the need for a better understanding of the confirmation and dynamics of DNA molecules in microchannels stems from lab-on-a-chip applications. In these applications, DNA molecules play a role in different contexts. Most prominently, DNA microarrays are often contained in a microfluidic chamber to increase the mass transfer rate to the reactive surface [14]. Microfluidic systems have been reported for extracting DNA from complex samples (see, e.g., Chung et al. [15]). Moreover, in many assays it is preferable to increase the DNA concentration in order to increase reaction rates or to lower detection limits. A number of different methods have been reported to pre-concentrate DNA in microchannels [16,17]. A classic application is the size separation of DNA, for which a number of microfluidic and nanofluidic methods exist [18,19,20]. Last but not least, significant effort has been spent on designing microfluidic devices for DNA amplification [21].

## 5. Experimental Strategies

Studying the dynamics of a single polyelectrolyte molecule was made possible in the past decades by a variety of techniques including magnetic tweezers, optical tweezers and atomic force microscopy, as well as techniques involving hydrodynamic flow or electric fields [22,23,24,25,26]. The first single molecule visualization was done by Yanagida et al. [27], where they recorded the conformations of T4 DNA molecules by epifluorescence microscopy. After that, a number of studies have been conducted with different techniques (using optical traps [28,29], magnetic beads [30] or a glass needle [31]) under different types of external forces on single DNA molecules. Related to the thematic scope of this review article, also single surface-tethered DNA molecules under external forces were studied in microchannels using fluorescence microscopy [24,25,26]. Moreover, single-molecule visualization was also utilized in studying DNA-protein interactions [32]. Apart from gaining a fundamental understanding of polyelectrolytes, single-molecule analysis finds its potential application in next-generation DNA sequencing methods. A comprehensive review of different next-generation sequencing techniques was given by Metzker [33]. In many of these techniques, DNA molecules are stretched and analyzed subsequently. For example, in direct linear analysis, the DNA molecules are stretched and analyzed by labeling the molecules with sequence-specific fluorescent probes [34]. The development of fluorescence microscopy techniques such as epifluorescence microscopy, total internal reflection fluorescence microscopy (TIRFM), confocal microscopy (CM), fluorescence recovery after photo-bleaching (FRAP), Förster or fluorescence resonance energy transfer (FRET) has substantially contributed to the field of single molecule studies.

The order of magnitude of the stretching force to deform a long-chain DNA molecule is $\frac{k_{B}T}{l_{p}}∼0.1$ pN. In magnetic tweezer experiments [35], the ends of the DNA molecules are attached to magnetic beads via non-covalent biotin-avidin or biotin-streptavidin linkages, after which the magnetic beads are manipulated by external magnets. The range of force achievable in this case is typically 0.01–10 pN. A larger force (0.1–100 pN) can be achieved by optical tweezers [36]. In optical tweezer experiments, a transparent micro-bead of refractive index higher than the liquid is trapped by a laser beam at a position near the focus. Attaching the ends of a DNA molecule to these trapped microbeads enables manipulation of the molecule and measuring of the deformation force acting on the molecule.

In case of a much larger force requirement (10–10,000 pN), atomic force microscopy (AFM) can be used [37]. In AFM, a cantilever of a known force constant is scanned over a DNA sample. A DNA molecule attached between the surface and the tip of the cantilever experiences a deformation force due to the bending of the cantilever. Thus, the elasticity of the molecule can be inferred by measuring the reflection of a laser at the cantilever.

Apart from applying an external force in the form of a bead-trapping mechanism or by an AFM cantilever tip, a DNA molecule can also be deformed by applying a hydrodynamic flow [24,25] or an electric field [26]. In these experiments, one end of the DNA molecule is immobilized, while the rest of the molecule is exposed to the external force. Immobilization of the end of the molecule is done by various methods. Smith et al. [28] attached one end of the molecule to a microbead using the biotin-avidin linkage and trapped the microbead at the end of a pipette tip by applying a suction. Very often, the end of the molecule is modified by an oligonucleotide that contains an end group to link it to a functionalized surface. Among the different linkages used for this purpose, thiol-on-gold (covalent) and biotin-avidin (non-covalent) bonding strategies are the most common [24,25].

Stretching of DNA molecules by uniform hydrodynamic flow was studied by Perkins et al. [24]. They attached a latex sphere to the end of a λ-DNA or concatenated λ-DNA molecule using the biotin-streptavidin bond. The latex sphere was held stationary against the flow by optical tweezers, and the extension of the molecule was visualized by fluorescence microscopy. Alternatively, Ladoux and Doyle [25] tethered λ-DNA or concatenated λ-DNA molecules to the surface of 200 μm deep microchannels and exposed the molecules to pressure-driven flow. Near the channel surface, the molecules effectively experienced a linear shear flow. Apart from the flow-induced manipulation, DNA molecules were also stretched under an electric field [26,38]. Ferree and Blanch [26] tethered the ends of DNA molecules to micro posts covered by gold at the top by the thiol-on-gold covalent linkage. After that, the molecules were stretched by applying a DC electric field. In subsequent sections, we discuss different tetehring mechanisms, visualization techniques and methods for applying external forces on a surface-tethered DNA molecule.

### 5.1. Tethering Mechanisms

**Figure 3:**
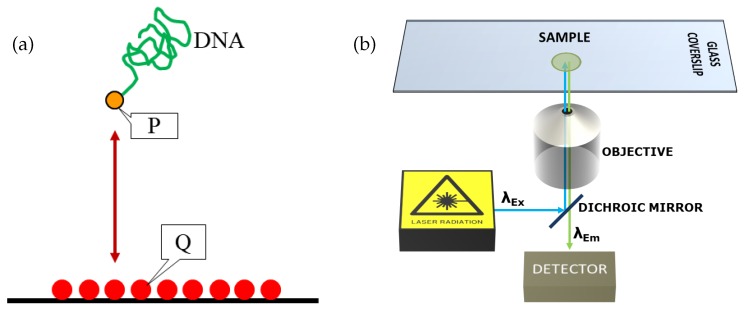
(**a**) Tethering strategy for DNA molecules. P and Q are a pair of binding entities. For example, P could be biotin, a thiol group or digoxygenin. Q could be streptavidin, gold or anti-digoxygenin-IgG, respectively. (**b**) Schematic diagram of a typical fluorescence microscopy setup.

Probably the most effective technique for single-molecule studies on the deformation and dynamics of long-chain DNA molecules is to tether a molecule to a solid surface at its one end and expose it to specific forces. Apart from the fundamental understanding of the molecular behavior, end-tethered DNA molecules find applications in the preparation of polymer-brush-covered surfaces for reducing friction, stabilization of colloidal dispersions or the fabrication of smart surfaces. Researchers have used different linkages for realizing the tethering of DNA molecules, e.g., streptavidin-biotin, thiol-on-gold, digoxigenin-anti-digoxigenin-IgG, mono-or-heterobifunctional PEG linkers etc. [39]. The basic tethering strategy is depicted in Figure 3a.

Streptavidin and biotin are proteins that form one of the strongest non-covalent bonds with a dissociation constant of ≈$10^{-14}$ M and a maximum force of ∼200 pN. [40] The common method is to immobilize the straptavidin on the surface, modify the end of the DNA molecule with biotin and bring the modified molecules in contact with the streptavidin-coated surface. Immobilization of streptavidin can be realized by incorporating reactive groups on the surface that can form a covalent bond with the amine or carboxyl moiety of streptavidin. For example, a silica surface can be treated with oxygen plasma to incorporate hydroxyl groups on the surface. Subsequently, the activated surface can be treated with aminopropyltriethoxysilane (for incorporating amine groups that react with the carboxyl moiety) or glycidyloxypropyltrimethoxysilane (for incorporating epoxy groups that react with the amine moiety). Modification of the end of a ds-DNA molecule can be done by ligating a biotin-modified single-stranded complementary oligonucleotide at the sticky end (i.e., the single-stranded overhang) of the DNA molecule in the presence of T4-DNA ligase [25]. Alternatively, one can make a careful selection of the collection of nucleotides which contains a biotin-modification at a specific nucleotide. After that, incorporation of the nucleotides at the sticky end of the long-chain ds-DNA produces an end-biotinylated molecule [41].

The other widely used end-tethering technique for DNA molecules relies on the ‘thiol-on-gold’ chemistry [42]. In that context, the end of a ds-DNA molecules is modified with a thiol (sulfhydryl, -SH) group by hybridizing a thiol-modified complementary single-stranded oligonucleotide at the sticky end of the DNA molecule [43]. The substrate has to be coated with a thin film of gold. This can be accomplished by the ‘lift-off’ technique [43]. Essentially, the substrate is first coated with a positive photoresist, which is then patterned and developed. After that a chromium layer followed by a gold layer are deposited. The chromium layer is used to increase the adhesion of the gold layer to the substrate. Subsequently, the photoresist layer and the overlaying metal layers are lifted off by a solvent.

Alternatively, the tethering of DNA molecules can be achieved by digoxygenin-anti-digoxygenin- IgG binding (dissociation constant ∼$1.2\times 10^{-9}$ M, maximum force ∼25 pN). Typically, a digoxygenin- modified nucleotide (obtained by an enzymatic polymerase chain reaction) is incorporated at the end of a DNA molecule. The corresponding antibody anti-digoxygenin-IgG is non-specifically adsorbed to a nitrocellulose-coated substrate [44]. The end of a DNA molecule can also be tethered to a solid surface using bi-functional polyetheylene glycol (PEG) chains containing functional moieties specific to the end-functional group of the DNA molecule and the functional group on the surface [45].

### 5.2. Visualization Techniques

One of the main challenges in single-molecule visualization is posed by the diffraction limit, i.e., the inability of an optical instrument working in far-field mode to resolve two objects separated by a distance less than approximately half the wavelength of light used to image the specimen. The cross-sectional diameter of a ds-DNA molecular chain is ∼2 nm, which is too small to be resolved with far-field optical imaging techniques. The visualization of DNA molecules in a fluid medium is mostly based on fluorescence microscopy [46]. In fluorescence microscopy, flourophores are attached to the sample molecules. The fluorescence of the fluorophores is then detected using a bandpass filter in combination with a photomultiplier detector or a CCD camera. A schematic diagram of this technique is shown in Figure 3b. This allows selective detection of the emission originating from the molecules, while background signals are largely suppressed. However, in far-field mode standard fluorescence microscopy techniques are subject to the diffraction limit as well. This means that the recorded image is a convolution of the real fluorescence intensity distribution with the point-spread function of the imaging device.

In epifluorescence mode (Figure 3a), the sample is usually excited with a laser beam. The complete volume in which the sample is contained (for example the microchannel) is illuminated by the beam. Therefore, the fluorescence signal originates from the focal plane of the microscope ($z=z_{0}$) as well as from regions above and below the focal plane. This makes imaging with depth resolution difficult to impossible. As an alternative, laser-scanning confocal microscopy can be used to image the full 3D structure of a specimen, bearing in mind the constraints due to the diffraction limit. In confocal imaging the image is obtained via a point-by-point scanning of the sample volume by the exciting laser beam. The out-of plane contributions of the signal are suppressed by passing the fluorescence light through a pinhole. This enables a submicron resolution both in plane and in *z*-direction. However, as a scanning technique, confocal microscopy is inherently slow. Usually, the time it would take to image the 3D structure of a polymer is significantly longer than the characteristic time for conformational changes of the molecule.

## 6. Mesoscopic Simulation Approaches

In this section, we describe the available simulation techniques to model a confined polyelectrolyte in terms of simple models. Over the last decades, simple coarse-grained simulation techniques like Langevin or Brownian Dynamics (LD and BD), Dissipative Particle Dynamics (DPD), Lattice Boltzmann methods (LB) or Multiparticle Collision Dynamics (MPCD) were often used as efficient methods to study flow phenomena on length scales from nanometers to micrometers. The reason for the rapid success of coarse-grained methods is the usage of simple potentials as well as the explicit consideration of stochastic motion. Due to coarse-graining and in combination with a stochastic dynamics approach, large amounts of molecular degrees of freedoms are integrated out, which results in shorter computation times when compared to atomistic models. However, in order to study the time evolution of both coarse-grained as well as atomistic models, Newton’s equations of motion are numerically integrated via a standard molecular dynamics (MD) scheme. Here, we briefly summarize the basic properties of the aforementioned methods, and focus explicitly on the benefits of the coupled LB/MD method.

### 6.1. Short Summary of Standard Mesoscopic Simulation Methods

As we briefly mentioned, the dynamic properties of soft matter like polymers in solution are dominated by hydrodynamic interactions. In consequence, a careful simulation study of polymer dynamics requires advanced techniques that are able to reproduce hydrodynamic behavior. Noteworthy, the intrinsic dynamics of the solvent usually occurs on shorter time scales when compared with the global dynamics of polymers and is thus often ignored. In order to compensate the missing interactions between the solvent molecules and the main solute, the introduction of a stochastic dynamics approach is recommended. As the most simple approach, Brownian Dynamics simulations (BD) usually exclude hydrodynamic interactions. Taking these interactions explicitly into account [47] remains computationally unsatisfying, due to the long range (1/r)-decay of the Rotne–Prager tensor [48,49]. Efficient coarse-grained model schemes of the liquid, so called mesoscopic simulation approaches, have been invented in the last decades to overcome this situation.

Several methods like the Lattice Boltzmann method (LB) [50,51,52], Dissipative Particle Dynamics (DPD) [53,54,55,56] and Multi-Particle Collision Dynamics (MPC) [57,58] are used as efficient Navier–Stokes solvers. Although the theoretical background of these methods is well understood, the lattice/off- lattice and thermal/athermal character impedes a general straightforward mapping between them. A general discussion of mesoscopic simulation methods in microfluidic devices can be found in recent reviews [59,60].

**Figure 4:**
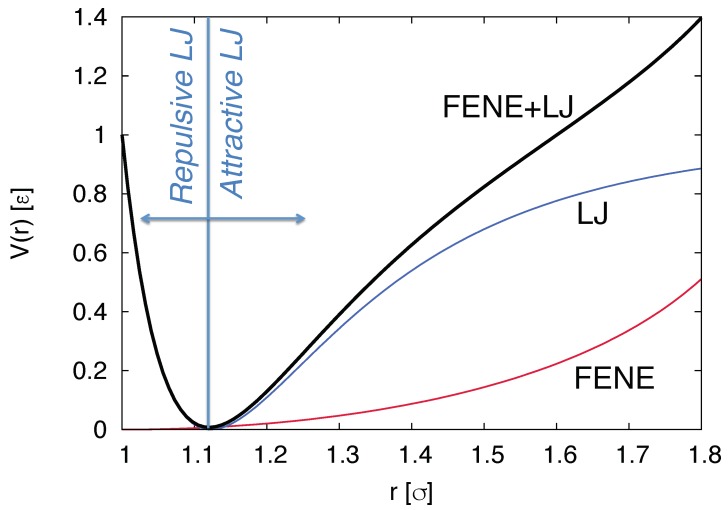
Schematic illustration of a modified Lennard–Jones potential (Equation (10), blue line), a finitely extensible nonlinear elastic (FENE) potential (Equation (11), red line) and the sum of both contributions (black line). The blue vertical line denotes the cutoff-distance $r_{c}$, where the Weeks–Chandler–Andersen potential as purely repulsive interaction vanishes for $r\geq r_{c}$.

In general all above-mentioned approaches and coupled LB/MD model the main solute explicitly and are thus particle-based. Therein polymers are modelled by means of pair potentials between point particles. If the long-ranged Coulomb interaction between charged monomers has to be taken into account, several methods exist that efficiently calculate the resulting forces [61,62,63,64,65,66]. Further interactions between molecules or fictitious atom groups in coarse-grained models are often approximated either by a Lennard–Jones [67] or by a purely repulsive Weeks–Chandler–Andersen potential [68]:

(10) $$V\left(r_{ij}\right)=4\epsilon \left[{\left(\frac{\sigma }{r_{ij}}\right)}^{12}-{\left(\frac{\sigma }{r_{ij}}\right)}^{6}+\frac{1}{4}\right],$$

where $r_{ij}$ is the distance between particle *i* and *j*, ϵ and σ are the characteristic energy and lengthscale. The bonds between adjacent monomers can be modeled via the finitely extensible nonlinear elastic (FENE) bond potential:

(11) $$V\left(r\right)=-\frac{1}{2}K\Delta r_{\max}^{2}\ln\left[1-{\left(\frac{r-r_{0}}{\Delta r_{\max}}\right)}^{2}\right],$$

in order to avoid entanglement effects, where *r* is the monomer-monomer distance, *K* is a force constant defining the stiffness of the bonded interaction, $\Delta r_{\max}$ the maximum elongation of the bond and $r_{0}$ the equilibrium elongation. Equations (10) and (11) are illustrated in Figure 4.

DPD and MPCD also rely on the use of coarse-grained spheres to model solvent beads. Stochastic movement of all species is then induced by the introduction of random forces for DPD, or a random angular displacement for MPCD. Both methods conserve the momentum and are Galilean invariant, which is a necessary prerequisite for hydrodynamic behavior. However, the introduction of solvent beads needs an increasing amount of computations for each time step, which is the main drawback of both methods when compared to coupled LB/MD. Due to this reason, we now explicitly outline the main principles of coupled LB/MD schemes in the context of soft matter simulations.

### 6.2. Lattice Boltzmann Method and Coupled LB/MD

To incorporate hydrodynamic interactions in the mesoscopic simulations, either a fluid is modeled explicitly by coarse-grained solvent molecules or the Navier–Stokes equation is solved on a continuum scale, e.g., via the Lattice Boltzmann method (LBM). Modeling the solvent explicitly increases the number of degrees of freedom in the simulation considerably. Thus, also the computational cost will be higher. Using a continuum scale hydrodynamics solver introduces less additional degrees of freedom and is therefore more efficient in terms of the computational effort.

LBM is a grid-based approach to solve the incompressible Navier–Stokes equation numerically. Advantages of this method are the ease of implementation and the possibility to treat complex boundary conditions with reasonable effort. In the LBM, a discretized version of the Boltzmann transport equation is solved. On every grid node and at each time step, a finite set of phase space densities of fictitious particles is prescribed. In comparison with the continuous Boltzmann equation, the phase space densities correspond to discretized number densities of probability distributions. The number of phase space densities depends on the dimensions of the problem domain and on the chosen model. The most commonly used lattice model for three dimensions is the so-called D3Q19 lattice with 19 phase space densities on every node. In every iteration of the algorithm, these densities first stream to the node they point to. After the streaming step a collision operator relaxes the populations to an equilibrium distribution. A collision operator may relax all modes of the populations with the same rate (single relaxation time (SRT)) or have multiple relaxation rates for different modes (multiple relaxation times (MRT)).

In order to couple MD and LBM and thus include hydrodynamic interactions in the simulations, Ahlrichs and Dünweg proposed a simple frictional coupling scheme [69,70]. According to this scheme, the force acting on the explicit particle, either a monomer bead or an ion, is proportional to the difference between the velocity of the particle $\vec{v}$ and the interpolated fluid velocity $\vec{u}$ at the particle position.

(12) $${\vec{F}}_{\mathrm{fluid}}=-\Gamma \left(\vec{v}-\vec{u}\right),$$

where Γ is a frictional constant.

The magnitude of the interaction energy of molecules in soft matter is usually comparable to the thermal energy. Thus, including thermal fluctuations in the simulations of such systems is crucial in order to get the correct static and dynamic interactions.

For the hybrid MD/LB simulation scheme the fluid itself and the MD particles have to be thermalized. For the thermalization of the MD particles it is important to conserve the momentum of the system. The thermalization of the Lattice Boltzmann fluid is realized by adding a stochastic term to the non-conserved modes [71,72]. A commonly used method for the particle thermalization is Langevin dynamics [73]. In order to conserve momentum, a counter force to the stochastic force of the MD particle has to be applied onto the LB fluid.

## 7. Results

The behavior of long-chain polymers is different in micro or nano-confinement as compared to that in an unconfined situation [22,74,75]. Shallow microfluidic channels are often used for manipulating long-chain polyelectrolytes for various applications. Such shallow channels facilitate the visualization of the molecules. In most of the experimental setups, DNA molecules are visualized by a high magnification and high numerical aperture objective. A typical value of the working distance of such objectives is about 200 μm. It is therefore advantageous to use shallow channels to be able to cover the entire height of a channel. In the following sections, the results on the deformation and relaxation of end-tethered polyelectrolytes in different degrees of confinement are summarized.

### 7.1. Strong vs. Weak Confinement

The effect of confinement on a polymer manifests itself by the interplay of three length scales: The characteristic dimension of the confining geometry (*h*), the radius of gyration of the polymer coil ($R_{g}$) and the persistence length of the chain ($l_{p}$). In weak confinement, i.e., when $h>>R_{g}$ and h>L, the dynamics of a tethered chain is dictated by the forces on the chain and the presence of the tethering surface, where *L* is the contour length of the chain. In the moderate confinement regime, i.e., when $R_{g}<h<L$, the confinement-induced change in the flow-profile (for flow-induced stretching) becomes important [41,76]. In strong confinement, i.e., when $l_{p}<h<R_{g}$, entropic stretching is observable and the chain can effectively be described by a succession of blobs of size *h* in a 2D (slit-like confinement) or 1D (cylindrical confinement) configuration. This means that the strong confinement scenario is qualitatively different from the other two scenarios. In all of these confinement regimes, the change in the effective drag coefficient of the molecule due to the presence of the tethering surface plays a key role in their dynamics. Here we summarize the studies on the stretching and relaxation dynamics of end-tethered polymers in unconfined or moderately confined situations, as characteristic for microchannels.

### 7.2. Stretching

An end-tethered polyelectrolyte stretches in different ways when exposed to hydrodynamic flow and electric fields. The stretching dynamics also depends on the geometric boundary conditions, i.e., whether the stretched molecule is in close proximity to a surface or not. In the former case, the hydrodynamic interactions among different segments of a chain are suppressed. The effects of the stretching forces on the deformation of a molecule are discussed in detail in the next sections.

#### 7.2.1. Under Hydrodynamic Drag

**Figure 5:**
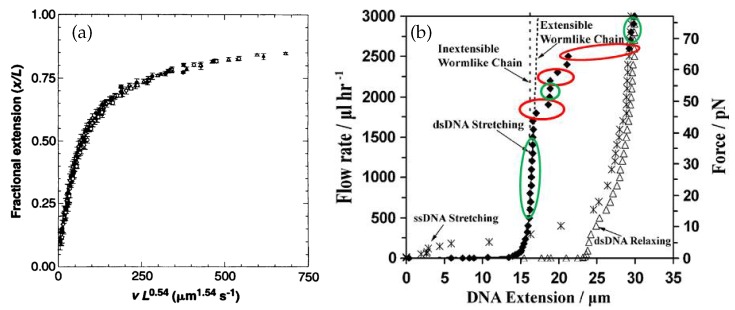
(**a**) Variation of the fractional extension of a DNA molecule with the similarity parameter $vL^{0.54}$, obtained from the experiments by Perkins et al. [24]. (**b**) Force-extension curves for single and double-stranded DNA molecules, obtained from the experiments by Wang and Lu [77]. The stretching transitions and plateaus are marked with red and green ellipses, respectively.

The behavior of a tethered polymer in uniform and shear flow has been studied both experimentally [24,25,41,77,78,79] and numerically [80,81,82,83,84]. The experimental studies report the validity of the worm-like chain model for describing the elasticity of the DNA molecules. Perkins et al. [24] characterized the stretching of end-tethered DNA molecules in a uniform flow field. They attached latex microspheres at the end of the DNA molecules and held the microspheres stationary using an optical trap. They found that the fractional extension (x/L) only depends on the combination $\eta vL^{0.54\pm 0.05}$, where η is the dynamic viscosity of the medium, *v* the flow velocity, *x* the extension of the molecule and *L* the molecular contour length (see Figure 5a). This universal functional dependency is observed owing to the fact that the combination $\eta vL^{0.54\pm 0.05}$ is directly proportional to the hydrodynamic drag on the chain which is responsible for the stretching. The fractional extension can, therefore, be expressed as a unique function of the stretching force (see Equations (1)–(4)). The exponent of *L* clearly indicates the presence of intra-chain hydrodynamic interactions even close to full extension (∼80%). Wang and Lu [77] observed multiple stretching transitions and plateaus for double-stranded and single-stranded DNA molecules tethered at one end to a microchannel surface and at the other end to 5 μm beads. The stretching force was mainly due to the hydrodynamic drag on the microbead. It was possible to describe the extension curves of the molecules by the extensible and inextensible worm-like chain models (see Figure 5b).

**Figure 6:**
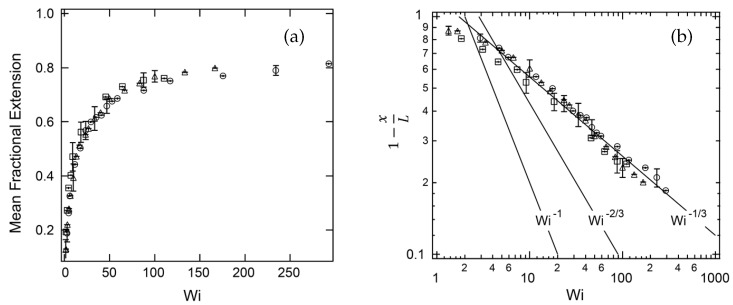
Experimental results obtained by Ladoux and Doyle [25]. (**a**) Mean fractional chain extension vs. Weissenberg number (Wi): L=18.9μm (squares), L=37.8μm (triangles), and L=56.7μm (circles). (**b**) Comparison of the experimental data to scaling laws resulting from the worm-like chain, freely-jointed chain and stem and flower model. The symbols are the same as in (**a**).

The dynamics of tethered DNA molecules on the surface of a microchannel was explored by Doyle et al. [78] using single-molecule experiments and Brownian dynamics simulations. The free end of an end-tethered chain undergoes a continuous recirculating motion when exposed to a shear flow. This phenomenon is known as the cyclic dynamics. Doyle et al. [78] observed a shear-induced cyclic dynamics of the surface-tethered molecules, due to the coupling of the flow-velocity gradient near the wall and thermal fluctuations. They investigated the stretching of surface-tethered long-chain DNA molecules in shear flow by single-molecule experiments and analytical scaling arguments. The channel height was 200 μm, which corresponds to an effectively unconfined situation. A Poiseuille flow was generated in the microchannel to impose an effectively linear shear flow on the DNA molecules of three different contour lengths (18.9 μm, 37.8 μm and 56.7 μm). They found that for a DNA molecule tethered to a surface in the strong stretching limit, the fractional extension scales like $\frac{x}{L}∼\left(1-{\dot{\gamma }}^{-1/3}\right)$, where $\dot{\gamma }$ is the shear rate at the wall (see Figure 6). In this limit, intra-chain hydrodynamic interactions are negligible [85]. It was found that the fractional extension is a unique function of the Weissenberg number, which is the product of the imposed shear rate and the longest relaxation time of the molecules.

**Figure 7:**
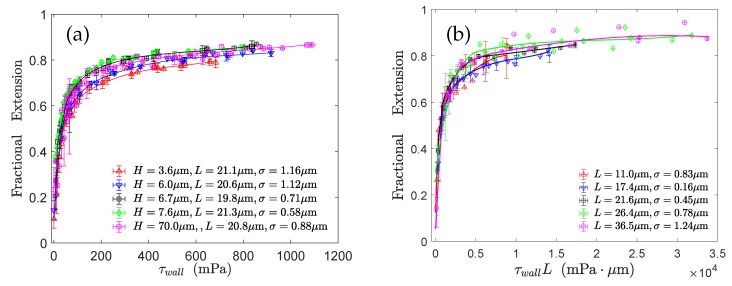
Experimental results obtained by Roy et al. [41] for the stretching of surface-tethered DNA molecules under weak confinement: (**a**) effect of the degree of confinement on the fractional extension and (**b**) validation of the unique functional relationship between the fractional extension and $\tau_{wall}L$.

**Figure 8:**
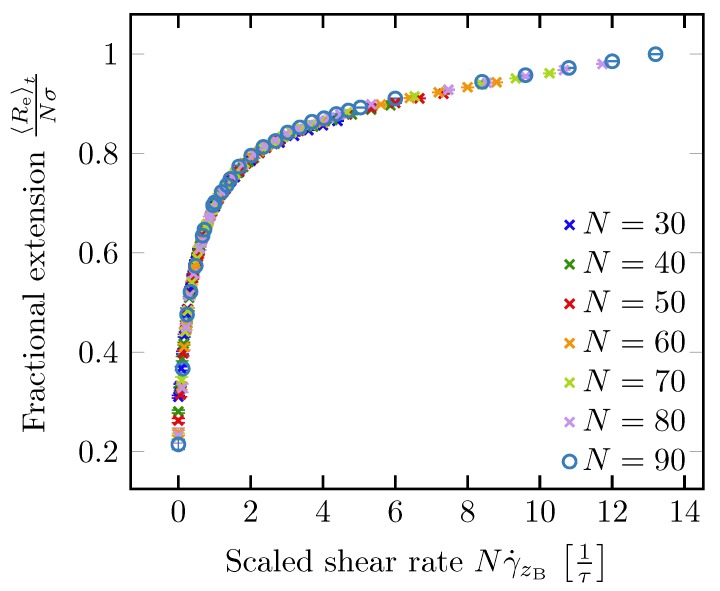
LB/MD simulation results for the fractional extension of a polymer as a function of the shear rate at the boundary ${\dot{\gamma }}_{z_{B}}$ multiplied with the number of monomers *N*. Taken from Ref. [86].

Lueth and Shaqfeh [79] visualized end-tethered DNA molecules under shear flow in a plane normal to the tethering plane and were able to reveal the details of the cyclic dynamics using theoretical, computational and experimental approaches. They characterized the cyclic dynamics by the autocorrelation function of the chain orientation angle. The power spectral density is the magnitude of the Fourier transform of the autocorrelation function. They found a maximum in the power spectral density function of the polymer orientation angle, confirming that the cyclic dynamics is quasiperiodic in nature. Roy et al. [41] studied the effect of confinement on surface-tethered DNA molecules using laser-scanning confocal microscopy and coarse-grained Lattice Boltzmann molecular dynamics simulations. The degree of confinement was moderate, i.e., the height of the microchannels was smaller than the contour length of the molecules, but larger than their radius of gyration. They only found a weak effect of the confinement on the stretching of the molecules. In shallower channels, the fractional extension is somewhat smaller (see Figure 7a). In addition, their results show that the fractional extension is a unique function of the product of the wall shear rate and the molecular contour length (see Figure 7b) which can be attributed to the suppression of intra-chain hydrodynamic interactions near a solid surface. LB/MD simulations of a tethered polymer in a Poiseuille flow confirmed this finding (see Figure 8) [86]. Szuttor et al. [86] found that the fractional extension is a unique function of the shear rate for different degrees of confinement up to a certain limit. In very narrow channels (channel height≈radius of gyration) the spatially varying shear rate of the fluid flow leads to a deviation from the master curve.

Gratton and Slater [83] investigated the stretching behaviour of a tethered polymer by means of molecular dynamics simulations. Their system consisted of a bead-spring polymer model with FENE bond potentials and two confining walls. To impose a Poiseuille flow they applied an external force onto the solvent particles. In consistency with the results obtained by Doyle et al. [78] they observe that the investigated freely-jointed chain’s fractional extension scales as ${\mathrm{Wi}}^{-\frac{2}{3}}$. Furthermore, they studied the aforementioned ‘cyclic dynamics’ by evaluating the cross-correlation between the end-to-end distance and the vector joining the anchor point and the center of mass of the polymer. They conclude that the cyclic motion of the chain can be described by four steps: (1) By thermal fluctuation the chain explores regions with higher flow rate, (2) the strong flow stretches the chain, (3) since the chain is tethered it gets pushed towards the wall, (4) since the flow rate is smaller at the wall it returns to a more relaxed state.

#### 7.2.2. Under Electric Fields

**Figure 9:**
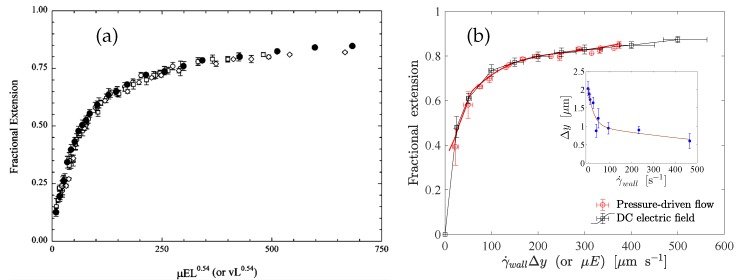
Experimental results demonstrating the electro-hydrodynamic equivalence principle (EHEP) for (**a**) end-tethered unconfined DNA molecules [26] and (**b**) end-tethered DNA molecules at the surface of shallow microchannels [92].

**Figure 10:**
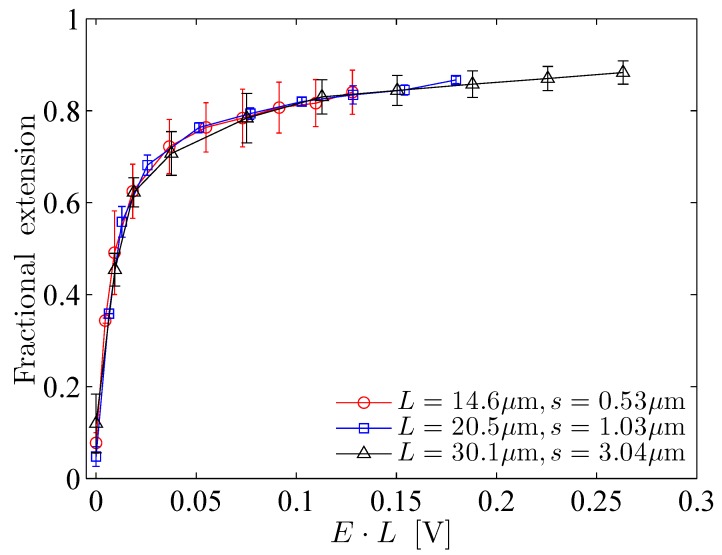
Fractional extension plotted as a function of the product of the applied electric field and the respective molecular contour length. The lines are guides to the eye.

The stretching of polyelectrolytes under electric fields has been extensively studied in the past, based on experimental [26], numerical [87,88,89,90] and theoretical methods [9,91]. This is especially important for understanding the electrophoresis of DNA molecules in free solution and in cross-linked gels. In the presence of counter-ions in the solution, a Debye layer forms around the charged backbone of the DNA molecules. The thickness of the Debye layer is approximately given by

(13) $$\lambda_{D}={\left(\frac{\epsilon_{0}\epsilon_{r}k_{B}T}{e^{2}\sum_{i}z_{i}^{2}C_{i}}\right)}^{1/2},$$

where $\epsilon_{0}$ is the vacuum permittivity, $\epsilon_{r}$ the dielectric constant of the solution, $k_{B}$ the Boltzmann constant, *T* the absolute temperature, *e* the elementary charge, $z_{i}$ the valance of the $i^{th}$ ionic species, and $C_{i}$ is the concentration of the $i^{th}$ ionic species. In free-solution electrophoresis (i.e., in the absence of a sieving matrix) and in the presence of counter-ions of a sufficiently high concentration, DNA molecules are free-draining, i.e., no net force is exerted that deforms the chains. This is often explained by the absence of intra-chain hydrodynamic interactions in the resulting electroosmotic flow (EOF) around the charged backbone. For sufficiently thin Debye layers, the electro-osmotic counterflow on the charged backbone locally cancels the flow generated by the motion of the polyelectrolyte. As a result, each segment of a DNA molecule experiences the same hydrodynamic drag force, and consequently, the electrophoretic mobility of the molecule becomes size-independent. Ferree and Blanch [26] studied the stretching of end-tethered ds-DNA molecules in an effectively unbounded fluid medium under a DC electric field. They tethered the ends of the molecules to the tops of microposts using a thiol-on-gold and biotin-neutravidin chemistry. They found that the fractional extension of the DNA molecules is a unique function of $\mu EL^{0.54}$, where μ is the electrophoretic mobility of molecules and *E* is the applied electric field strength (see Figure 9). Roy et al. [92] studied the stretching of surface tethered polyelectrolytes in a DC electric field under moderate confinement (i.e., H<L). They used λ-DNA and concatemers of λ-DNA as model polyelectrolytes and found that the fractional extension is a universal function of the product of the applied electric field strength and the molecular contour length (see Figure 10) in different degrees of confinement, while no significant dependence on the channel height was found. Their experimental results indicate that the universal functional relationship between the fractional extension and the product of the applied electric field strength and the molecular contour length is valid at least in the range $2.6\leq \frac{L}{h}\leq 8.4$.

When compared to the stretching in a uniform flow field, one can directly identify an equivalence in terms of the electric and flow parameters. This is known as the electro-hydrodynamic equivalence and discussed in detail in the next section.

Hickey et al. [93] investigated the importance of hydrodynamic interactions for the electrophoretic stretching behavior of a polyelectrolyte by means of hybrid MD/LB simulations. The system consists of a charged bead-spring polymer model with WCA interactions between monomers in a slit-like confined geometry. The polyelectrolyte was fixed on one end to one of the confining walls, and an external force on the charged polymer beads as well as on the charged salt ions was applied. In order to extract the influence of hydrodynamics they performed simulations in which the polymer beads are coupled to a Lattice Boltzmann fluid and compared them to Langevin dynamics simulations where the solvent is modeled implicitly. Concerning the stretching behavior, they observed that hydrodynamic interactions play a role in the following scenarios: (1) Intermediate electric field strengths, (2) high salt concentrations and (3) weak confinement. The fact that hydrodynamic interactions can play a role for high salt concentrations in this case was contrary to conventional wisdom. This can only be observed if the tethered object is larger than the salt induced Debye length [93]. In this scenario the large amount of coordinated ions implies an amplification of hydrodynamic interactions. Intermediate electric field strengths are necessary for hydrodynamic interactions to play a role due to the negligible electroosmotic flow along the chain for small fields. For very high electric field strengths the maximum elongation of the chain is limited by the contour length and the influence of the electroosmotic flow vanishes. Also, for low salt concentrations the electroosmotic flow is not concentrated around the chain and thus has a weak influence on the stretching. (3) can be explained by the well-known screening of hydrodynamic interactions in confinement [94].

#### 7.2.3. Electro-Hydrodynamic Equivalence

The electro-hydrodynamic equivalence principle (EHEP) is an outcome of the screening of hydrodynamic interactions in the EOF originating from the electric double layer around a polyelectrolyte chain [91,95]. The EHEP states that the stretching of a tethered polyelectrolyte molecule in an electric field is the same as in the case when the tethered molecule is exposed to a uniform flow velocity v=μE, where μ is the electrophoretic mobility of the molecule and *E* is the electric field strength. Despite this simple statement, the physical situation behind the EHEP for a surface-tethered polyelectrolyte is quite subtle, and it appears to be applicable only to a specific class of problems. First, to ensure the screening of hydrodynamic interactions, the Debye length needs to be smaller than the characteristic dimension of the polyelectrolyte chain. Secondly, a charged polyelectrolyte generates a local electroosmotic flow (EOF). The resulting flow field in the entire domain depends on the location of the boundaries (for example the channel walls), and a pressure-driven counterflow is generated due to the local nature of the source of the EOF. This situation is similar to the situation where only a part of a microchannel surface is charged. The charged part of the channel wall contributes to the EOF whereas the uncharged part does not, and as a consequence, a pressure-driven counterflow is generated as a reaction to the EOF. In this analogy, the tethered polyelectrolyte is equivalent to the part of the channel wall which is charged. It is then quite obvious that the hydrodynamic interaction among different segments of a polyelectrolyte chain will no longer be screened even if the Debye layer is sufficiently thin. A tethered polyelectrolyte in an electric field, therefore, gets stretched due to the pressure-driven flow that builds up as a reaction to the EOF around the charged backbone. Despite all of these limitations and subtleties, the EHEP has been confirmed experimentally for end-tethered polyelectrolytes in effectively unconfined [26] and confined [92] scenarios. Figure 9 depicts the corresponding results for (a) effectively unconfined and (b) surface-tethered long-chain ds-DNA molecules. When unconfined, an end-tethered polyelectrolyte stretches in the same way when the condition μE=v is satisfied. When a polyelectrolyte molecule is tethered to the surface of a microchannel, the molecule stretches in the same way under pressure-driven flow and under an electric field when the condition $\mu E=\dot{\gamma }\delta y$ is satisfied. The fractional extension is a unique function of μEL (or $\dot{\gamma }\Delta y$), where Δy is the distance normal to the surface sampled by the molecules due to transverse fluctuations.

**Figure 11:**
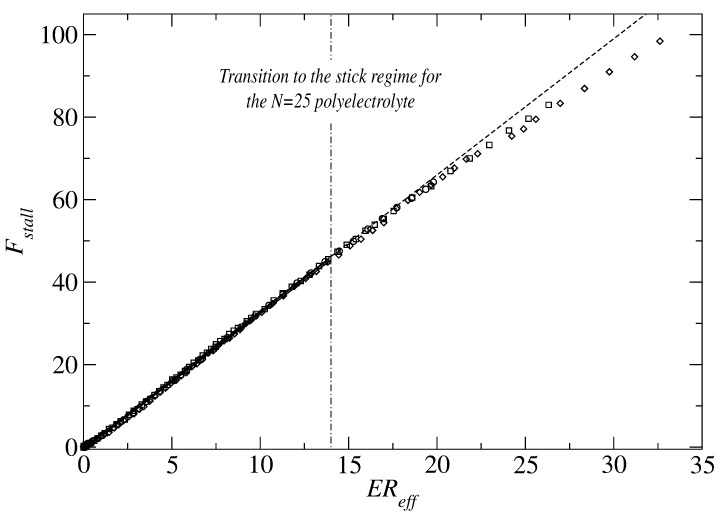
Stall force on a tethered model polyelectrolyte as a function of electric field times the ‘effective‘ hydrodynamic radius. Taken from Ref. [96].

In a simulation study by Betrand et al. [96] the predictions by Long et al. [91] are verified by investigating the force needed to keep a model polyelectrolyte tethered under the action of an externally applied electric field. As shown in Figure 11, the simulation data shows that the stall force is essentially proportional to the electric field and the effective hydrodynamic radius that accounts for the deformation of the polyelectrolyte: $R_{\mathrm{eff}}=\frac{R_{H0}}{R_{g0}}R_{g}$, where $R_{H0}$ is the equilibrium hydrodynamic radius, $R_{g0}$ the equilibrium radius of gyration, and $R_{g}$ is the radius of gyration of the deformed polyelectrolyte. Thus, their simulations support the hydrodynamic equivalence principle proposed by Long et al. [91].

### 7.3. Relaxation Dynamics

Before the development of single molecule visualization techniques, experimental methods like birefringence measurements, light scattering measurements and intrinsic viscosity measurements were used to estimate the relaxation time of polymer chains [97,98,99,100]. However, in these traditional methods, the dynamic properties of a polymer have to be inferred from the average properties of a macroscopically large number of molecules. It is also challenging to obtain a solution with a sufficiently narrow size distribution of the polymer molecular mass. In the past decades, however, researchers developed techniques allowing to study the relaxation of a single chain. In general, these methods capture the time evolution of the extension of an initially stretched chain towards its equilibrium conformation and allow inferring the relaxation time from that. The chain is initially stretched by an external force, after which the force is switched off to allow the molecule to relax. An uncharged chain can be stretched using hydrodynamic flow [101], whereas a polyelectrolyte can also be stretched by an electric field [38].

In order to estimate the relaxation time from the recoiling of the molecules, the stress on the chain can be represented as [102]

(14) $$s\propto \langle xF(x)\rangle ,$$

where *x* is the extension of the chain and *F* is the applied force. In the limit of small extensions (i.e., $\frac{x}{L}\leq 0.3$ [101], where *L* is the contour length ) the elasticity of the chain can be represented by Hooke’s law as [6]

(15) F(x)=kx,

where $k=\frac{3k_{B}T}{2l_{p}L}$, $l_{p}$ is the persistence length and *L* is the molecular contour length. The stress on the chain, therefore, becomes $s\left(t\right)\propto \langle x(t)x(t)\rangle$, where *t* is the time. The variation of $\langle x(t)x(t)\rangle$ with *t* is then fitted to a single-exponential decay function in the terminal-relaxation regime as

(16) $$\frac{\langle x(t)x(t)\rangle }{{\langle L\rangle }^{2}}=A\exp\left(-\frac{t}{\tau }\right)+B$$

to determine the longest relaxation time τ, where *A* and *B* are fit parameters. The numerical value of the longest relaxation time for double-stranded DNA molecules obtained by this method agrees with rheological measurements [76,103] and is nearly the same (within 10%) as the value obtained from numerical simulations where the longest relaxation time is determined from the temporal decay of the autocorrelation function of the end-to-end vector of a chain [104,105].

**Figure 12:**
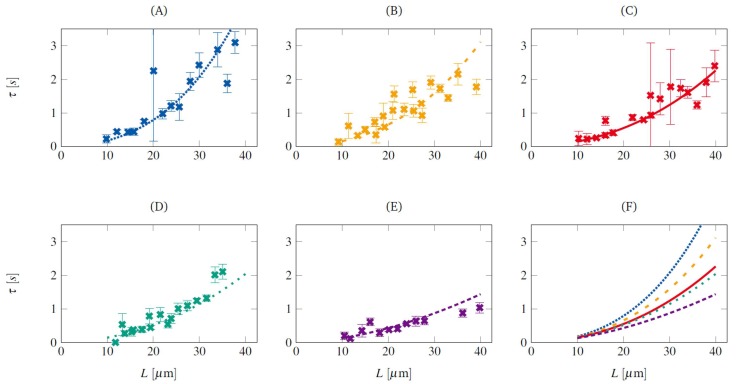
Experimental results on the relaxation of long-chain ds-DNA molecules under moderate confinement [106]. Each sub-plot shows the variation of the longest relaxation time with the molecular contour length, confined in microchannels of height (**A**) 2.5 μm, (**B**) 3.6 μm, (**C**) 6 μm, (**D**) 7.6 μm and (**E**) 70 μm. Sub-plot (**F**) shows the comparison of the relaxation time in different degrees of confinement.

The relaxation of ds-DNA molecules was first studied by Perkins et al. [101]. The molecules were tethered to 1 μm beads, manipulated by optical tweezers. The inverse Laplace transform of the time evolution of the end-to-end distance of the relaxing molecules exhibited distinct peaks, corresponding to different relaxation modes. It was found that the longest relaxation time increases with the molecular contour length as $\tau ∼L^{1.66\pm 0.10}$. The relaxation of DNA molecules, tethered to the surface of a microchannel in an effectively unconfined situation was measured by Ladoux and Doyle [25]. Their main objective was to normalize the shear rate imposed on the stretched molecule by the longest relaxation time and obtain the corresponding Weissenberg number Wi. The effects of moderate confinement on the relaxation time of ds-DNA molecules were studied by Hartmann et al. [106] using single-molecule experiments and LB/MD simulations. The DNA molecules were tethered to the surface of shallow (height smaller than contour length of the DNA molecules) microchannels. Their results revealed that the longest relaxation time of the molecules increases with increasing degree of confinement (see Figure 12), which can be attributed to the increased drag coefficient of the molecules due to the presence of nearby solid walls. A phenomenological scaling equation, given by $\tau =2.5\times 10^{-3}\;\cdot \;L^{1.71+0.83\;\cdot \;\tanh\left(\frac{R_{g}}{h}\right)}$ was obtained. Here τ, *L*, $R_{g}$ and *h* are the longest relaxation time (measured in seconds), molecular contour length (measured in μm), radius of gyration (measured in μm) and channel height (measured in μm), respectively.

## 8. Perspectives

Single-molecule experiments have significantly contributed to understanding the conformation and dynamics of polyelectrolytes, specifically DNA molecules. Results concerning the conformations and dynamics of surface-tethered polyelectrolytes, as obtained from single-molecule experiments and mesoscopic molecular dynamics simulations, were reviewed in this paper. Different from strong confinement, moderate confinement does not induce an entropic stretching of the polyelectrolyte. However, a number of somewhat more subtle effects occur that have been reported in this article. The results obtained in the moderate confinement regime are relevant in a number of different areas, since polymers tethered to microchannel surfaces have found a number of different applications, for example in context with surface functionalization or DNA microarrays. The studies reported in this article could be extended in different directions. It would be interesting to see how surface-tethered polymers react under transient forcing, for example a time-harmonic force. An extension that suggests itself is to study the interaction of neighboring surface tethered polymers under an applied force. Last but not least, the studies that have been mostly limited to linear polymers could be extended to branched polymers.
